# Mississippi Dental Association

**Published:** 1907-07

**Authors:** 


### MISSISSIPPI DENTAL ASSOCIATION.

The fourteenth annual meeting of the Mississippi Dental Association, held in Meridian, Miss., May 28th, 29th and 30th, proved to be the best in the history of the Association. A great many young men were received as members and the membership is double what it was three years ago.

The social feature was a banquet tendered the Association by the Meridian Dental Society, and was presided over by Dr. C. J. Washington, of Memphis, Tenn., as toastmaster, and many well chosen toasts were responded to by the members present.

The following officers were elected: President—Dr. L. A. Smith, Port Gibson; First Vice-Pres.—Dr. J. F. Brunson, Meridian; Second Vice-Pres.—Dr. C. F. Boger, Natchez; Secretary—Dr. E. Douglas Hood, Tupelo; Cor. Sec.—Dr. L. B. Price, Corinth; Treasurer—Dr. C. C. Crowder, Kosciusko.

The Association will meet in Jackson next year.

